# Asymmetric myocardial thickening in aortic stenosis

**DOI:** 10.1186/1532-429X-17-S1-Q49

**Published:** 2015-02-03

**Authors:** Calvin W Chin, Emily N Yeung, Anoop S Shah, Scott Semple, Maria Koo, Nicholas Mills, David Newby, Marc R Dweck

**Affiliations:** 1 Centre for Cardiovascular Science, University of Edinburgh, Edinburgh, UK

## Background

Asymmetric wall thickening has been observed in aortic stenosis (AS) but the clinical importance is poorly understood. We hypothesized this pattern was associated with advanced remodeling and worse outcomes.

## Methods

Left ventricular volumes, wall thickness and mass were assessed in 166 patients (70 [64, 76] years; 69% males) with cardiovascular magnetic resonance. Diffuse myocardial fibrosis was assessed using myocardial T1 mapping (partition coefficient, λ). In the absence of infarction, asymmetric wall thickening was defined as myocardial thickness ≥13 mm and opposing wall thickness ratio ≥1.5. High-sensitivity cardiac troponin I (cTnI) and brain natriuretic peptide (BNP) concentrations were used as markers of myocardial injury and decompensation, respectively. Aortic valve replacement and all-cause mortality were assessed at 1 year.

## Results

Compared to patients with concentric wall thickening (n=69), those with asymmetric pattern (n=43) had increased diffuse myocardial fibrosis (λ values 0.48±0.04 versus 0.46±0.04, respectively; P=0.04) despite similar age, sex, systolic blood pressure (SBP), and left ventricular mass index (LVMi; Table 1 and Panel A; all P>0.10). Plasma cTnI and BNP concentrations were also increased independent of age, sex, SBP, AS severity and LVMi (both P<0.01; Panels B and C). Patients with asymmetric pattern had worst outcomes compared to those with concentric thickening and normal wall thickness (log-rank P<0.0001; Panel D).

## Conclusions

In aortic stenosis, asymmetric wall thickening is associated with ventricular decompensation and a worse prognosis.

## Funding

The study is supported by the British Heart Foundation.

**Figure 1:**
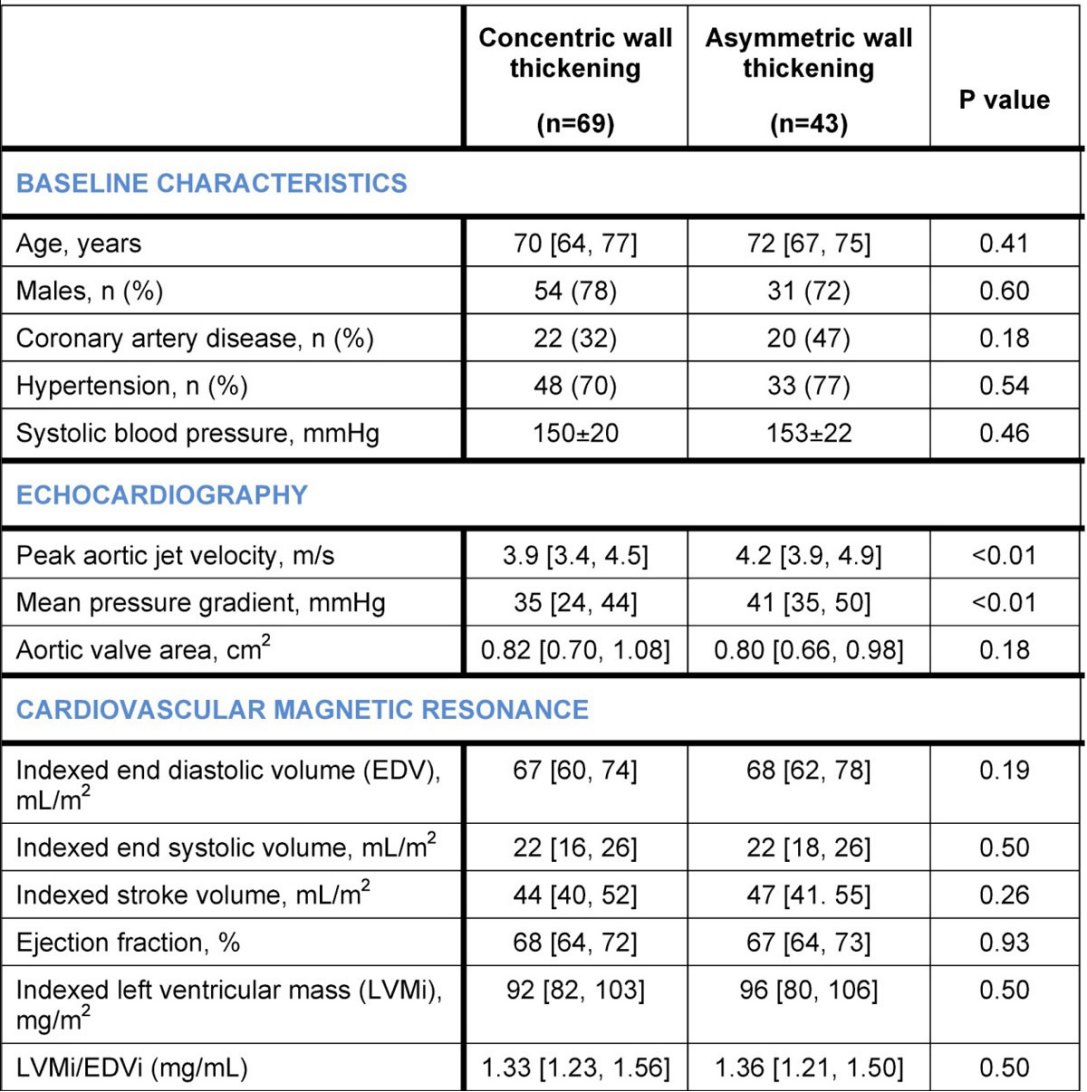
Baseline characteristics of patients with concentric and asymmetric wall thickening.

**Figure 2:**
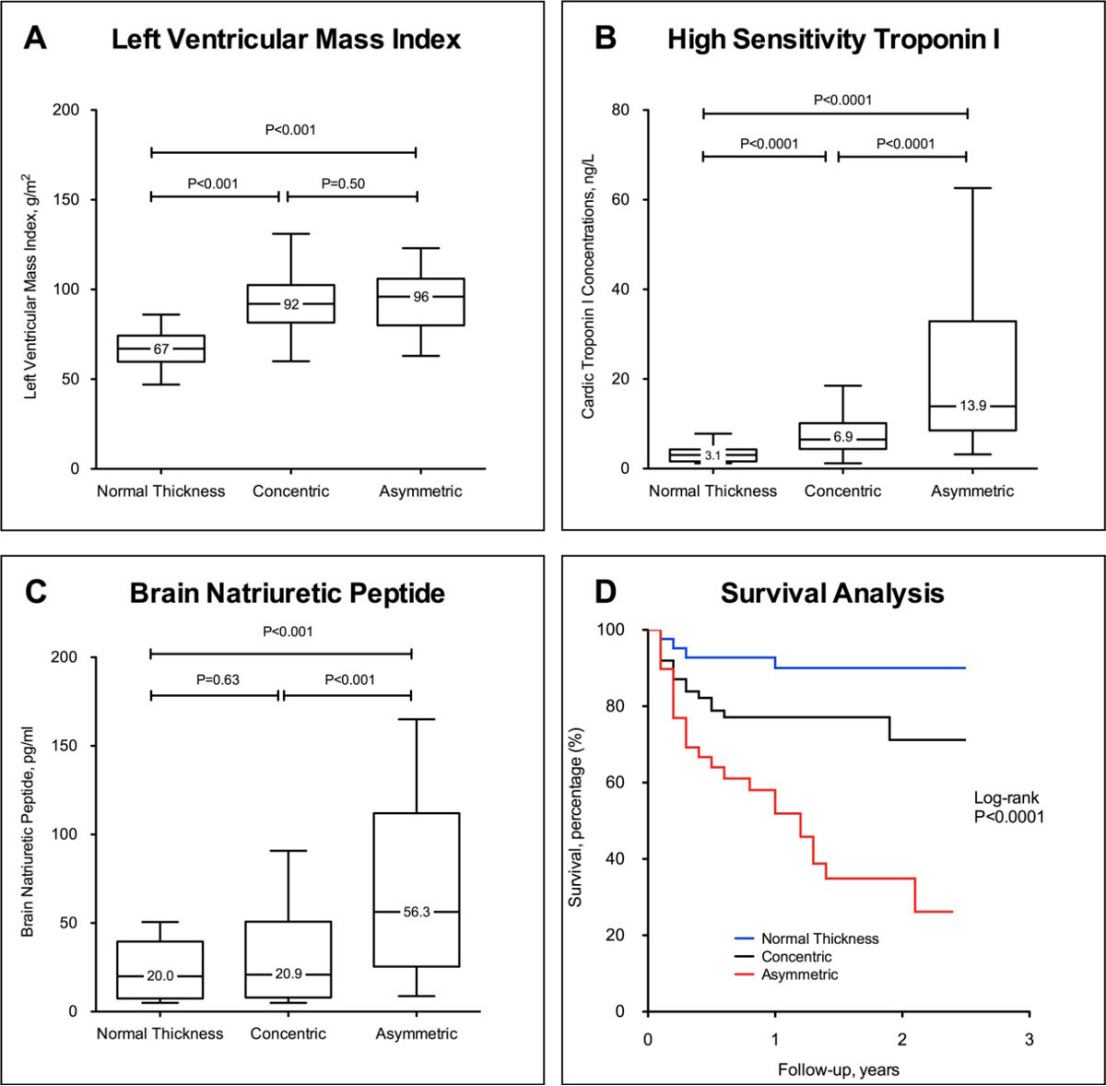
Compared to patients with concentric wall thickening, patients with asymmetric wall thickening had smilar left ventricular mass index (A) but elevated high-sensitivity troponin I (B) and brain natriuretic peptide concentrations (C). Importantly, patients with asymmetric wall thickening had worst outcomes compared to those with normal and concentric wall thickening (D).

